# Continuation of sedative infusions during palliative extubation for refractory status epilepticus in hepatic encephalopathy: A case report

**DOI:** 10.1177/2050313X261468910

**Published:** 2026-07-18

**Authors:** Liam D. Ferreira, Shailaja Hayden

**Affiliations:** 1 Department of Neurology, The University of Texas at Austin Dell Seton Medical Center, TX, USA; 2 Department of Pulmonology and Critical Care, The University of Texas at Austin Dell Seton Medical Center, TX, USA

**Keywords:** critical care/emergency medicine, neurology, palliative medicine

## Abstract

Refractory status epilepticus complicating hepatic encephalopathy poses unique palliative challenges as standard extubation protocols typically reduce or discontinue sedative infusions, risking immediate seizure recurrence and distress. We report a 53-year-old man with end-stage cirrhosis and hepatocellular carcinoma who developed hepatic encephalopathy and refractory status epilepticus precipitated by small bowel obstruction. Despite ammonia-lowering therapy and multiple antiseizure medications, seizures remained refractory. Electroencephalography confirmed status epilepticus despite escalating therapy. Given prohibitive surgical risk and poor prognosis, the multidisciplinary team and patient’s family elected to withdraw life-sustaining treatments, requesting he not die with an endotracheal tube in place. He underwent palliative extubation while continuing propofol and midazolam infusions to suppress seizure activity and ensure comfort. He died peacefully without visible seizure activity. Although limited by a single patient, this case provides a practical example of a rarely described palliative strategy: continuing sedative infusions during palliative extubation to prevent agonal seizures in refractory status epilepticus.

## Introduction

Hepatic encephalopathy (HE) is a neurological complication of hepatic insufficiency and portosystemic shunting, characterized by altered mental status, neuromuscular dysfunction, and, in severe cases, cerebral edema.^1,2^ In its most severe form, HE can progress to coma and refractory status epilepticus (RSE).^2^ Severe hyperammonemia is central to the pathophysiology of HE-associated seizures: ammonia crosses the blood-brain barrier, promotes neuronal excitotoxicity, impairs inhibitory GABAnergic neurotransmission, and induces astrocyte swelling.^2,3^ These changes collectively lower the seizure threshold and promote epileptiform activity.^3^ While metabolic precipitants such as infection and gastrointestinal bleeding are common, mechanical causes like small bowel obstruction (SBO) are infrequent but clinically significant.^4^

Palliative extubation refers to the removal of an endotracheal tube and withdrawal of mechanical ventilation when life-sustaining support is no longer consistent with the patient’s goals of care.^5^ Palliative extubation in patients with active RSE presents a unique clinical dilemma. Standard protocols involve reducing or discontinuing sedative infusions to allow for spontaneous respirations. However, this risks precipitating agonal seizures and significant distress at the end of life.^5,6^ The ethical challenge to balance effective symptom control against the risk of hastening death makes these situations among the most challenging encountered in palliative care.^5^ To our knowledge, the deliberate continuation of intravenous (IV) anesthetic infusions specifically to prevent seizures during palliative extubation has not been previously described in published literature.

This case focuses on the decision-making surrounding palliative extubation in a patient with RSE who required ongoing IV anesthetic infusions, with an approach that prioritized seizure control for comfort-focused care at the end of life.

## Case description

A 53-year-old man with alcohol-related cirrhosis (Child-Pugh Class C) and metastatic hepatocellular carcinoma presented to an outside hospital with a 10-day history of worsening abdominal pain and nausea. He had no documented prior episodes of HE or seizures. He was found to have a SBO caused by a right-sided diaphragmatic hernia and subsequently transferred to our hospital on the same day given the complexity of the case. After evaluation by multiple surgical teams (foregut, surgical oncology, and thoracic surgery) during the first days of his hospitalization, he was deemed not an operative candidate given prohibitive perioperative mortality risk.

On day 4 of admission, the medical ICU team was called to bedside due to worsening mentation and increasing oxygen requirements. The patient was found to be completely obtunded with a serum lactate of 10 mmol/L (up from 7 mmol/L earlier that day) and was emergently intubated. He was mechanically ventilated and underwent chest tube placement for a large pleural effusion. He developed worsening encephalopathy, acute hypoxemic respiratory failure, acute kidney injury, and extreme hyperammonemia (601 μmol/L). His family agreed to a time-limited trial of critical care. Hyperammonemia was treated with lactulose enemas (200 g every 4 h) and continuous renal replacement therapy; rifaximin was held given his nil per os status.

On the evening of day 4 of admission, the patient was noted to have new epileptiform movements of the right arm. Neurological examination also revealed gaze deviation to the right with sluggish pupils, prompting emergent evaluation. He was administered 2 mg IV midazolam and propofol was titrated from 30 to 40, then to 50 µg/kg/min. However, the patient became hypotensive (blood pressure reaching a nadir of 40s/20s mmHg), which prompted discontinuation of propofol and initiation of norepinephrine. Propofol was subsequently reinitiated at 20 µg/kg/min and midazolam was started at 5 mg/h. A loading dose of levetiracetam 4 g IV was administered, followed by a maintenance dose of 750 mg IV twice daily. Lacosamide was loaded at 200 mg IV and continued at 100 mg IV every 12 h. Ketamine infusion was initiated at 20 µg/kg/min and subsequently weaned. Midazolam was uptitrated to a maximum of 40 mg/h, then reduced to 30 mg/h. Propofol was maintained at 20 µg/kg/min.

Continuous electroencephalography (cEEG) monitoring was initiated upon ICU admission and maintained throughout the hospitalization to guide antiseizure therapy and assess eligibility for medication weaning. The cEEG initially showed rhythmic epileptiform activity and absent spontaneous reactivity, followed by diffuse voltage attenuation and persistently absent reactivity following escalation of antiseizure therapy. Despite partial electrographic improvement, the overall background pattern remained severely abnormal, and downscaling of infusions was not feasible without recurrence of epileptiform activity, as confirmed by cEEG.

Within 48 h of ICU admission, the family and critical care team agreed further aggressive interventions would be medically non-beneficial. They jointly decided to transition his care to comfort measures only. The family expressed that they did not want their loved one to die with an endotracheal tube in place. Following multidisciplinary discussions involving critical care, neurocritical care, and palliative care teams, and after thorough conversation with the family, vasopressors were withdrawn and the patient underwent palliative extubation while continuing infusions of propofol (20 µg/kg/min) and midazolam (30 mg/h) to suppress seizure activity and ensure comfort during the terminal phase. He died peacefully without visible seizure activity approximately 1 h after extubation. Clinical timeline is also shown in Table 1.

**Table 1.** Clinical timeline.

Day	Events
Hospital day 1	Presentation with 10-day history of abdominal pain, nausea, and vomiting. Admitted for SBO secondary to right-sided diaphragmatic hernia
Hospital days 1–3	Evaluation by multiple surgical teams (foregut, thoracic, surgical oncology) who deemed patient not an operative candidate
Hospital day 4 (morning)	ICU team called to bedside for worsening mentation and hypoxia. The patient was found completely obtunded; lactate 10 mmol/L. Emergent intubation, mechanical ventilation, and chest tube placement. Time-limited trial of critical care agreed upon with family
Hospital day 4 (night)	New right arm epileptiform movements. Neurology emergently consulted. Midazolam 2 mg IV given; propofol uptitrated (30 → 40 → 50 µg/kg/min). Patient became hypotensive (BP 40s/20s mmHg); propofol stopped, norepinephrine started (max 0.43 µg/kg/min). Propofol reinitiated at 20 µg/kg/min. Midazolam infusion started at 5 mg/h. IV levetiracetam 4 g load → 750 mg BID. IV lacosamide 200 mg load → 100 mg q12 h. Ketamine infusion started at 20 µg/kg/min
Hospital day 5 (morning)	Midazolam titrated to maximum 40 mg/h, then weaned to 30 mg/h. Ketamine weaned. Diffuse voltage attenuation persisted. Propofol maintained at 20 µg/kg/min Agreement that further aggressive intervention was not medically beneficial. Transition to comfort measures only. Family expressed wish for patient not to die intubated
Hospital day 5 (evening)	Vasopressors withdrawn. Palliative extubation performed with continuation of propofol (20 µg/kg/min) and midazolam (30 mg/h). Patient died peacefully without visible seizure activity approximately 1 h after extubation

SBO: small bowel obstruction.

## Discussion

This case is notable for the deliberate continuation of propofol and midazolam infusions specifically to control RSE during palliative extubation. Palliative extubation is the removal of an endotracheal tube and withdrawal of mechanical ventilation when such support is no longer aligned with the patient’s goals.^5^ It aims to alleviate suffering and allow a more natural dying process. However, it carries the risk of increasing suffering, most commonly via respiratory distress or pain, if not done well. Palliative extubation protocols typically involve titration of opioids and sedatives to minimal effective dose to relieve dyspnea and distress, often reducing or discontinuing sedative infusions to allow spontaneous respiration.^5^ Although not currently standard of care, some authors have argued that palliative general anesthesia should be considered during palliative extubation of unresponsive critically ill patients, to minimize the risk of unrecognized suffering at the end of life.^6,7^ The treatment of RSE has been studied and documented in the literature.^8^ However, to our knowledge, the deliberate continuation of infusions originally titrated for seizure suppression, specifically through and beyond palliative extubation, has not previously been described, and institutional protocols to guide and document such decisions remain largely absent. In this patient, cessation of propofol and midazolam infusions carried a high risk of seizure recurrence, causing significant distress to both the patient and family in the final hours of life. By maintaining these infusions, effective seizure suppression was achieved while still honoring the goals of palliative extubation.

The continuation of propofol and midazolam in this case was not palliative sedation undertaken to deliberately reduce consciousness as an end goal, but rather the preservation of an already established seizure-suppression regimen. Both agents had been titrated for antiseizure purposes prior to the transition to comfort-focused care, and no dose escalation was made for the purpose of hastening death. The intended effects were seizure suppression and comfort, while the foreseeable but unintended risk of hastening death through respiratory depression was an accepted consequence of preserving an existing, clinically necessary regimen. Transparent multidisciplinary deliberation and family-centered communication were central to ensuring this approach remained aligned with the patient’s values and the family’s wishes.

The concurrent use of propofol and midazolam in a patient with end-stage liver disease warrants further discussion. Midazolam undergoes extensive oxidative metabolism and glucuronidation, and its clearance is significantly impaired in cirrhosis, resulting in prolonged sedation from accumulation of active metabolites.^9,10^ In this patient, midazolam was initiated as part of the sequential RSE treatment algorithm. Ketamine was added at a point of escalation as a mechanistically distinct complementary agent with NMDA receptor antagonism, rather than as a substitute for midazolam.^11,12^ The clinical team judged that reduction in GABAnergic tone carried a high risk of breakthrough activity given the persistent electrographic epileptiform discharged on cEEG. Once the goals of care quickly transitioned to comfort measures only, the prolonged sedative effect of midazolam was no longer a clinical concern and in fact contributed to the comfort-focused intent of the palliative plan.

Propofol-related infusion syndrome (PRIS) – a rare but life-threatening complication characterized by metabolic acidosis, arrhythmias, and renal failure – was a recognized concern given the dose and duration of propofol use.^13,14^ Serial serum lactate, creatine kinase, and triglyceride levels were monitored throughout the ICU admission. No clinical or biochemical markers consistent with PRIS were identified. The short overall duration of high-dose propofol infusion prior to transition to comfort care mitigated the cumulative risk.

Once goals of care transitioned to comfort measures only, the infusions, initially started for aggressive seizure control, naturally evolved into a primarily palliative intervention. By maintaining these agents through and after extubation, effective suppression of seizure activity was achieved, with the overarching objective of dignity and comfort at the end of life.

This approach required careful thought to balance optimal symptom control against the foreseeable risk of hastening death. Multidisciplinary consensus and transparent family-centered communication were essential to ensure the plan reflected the patient’s overall goals of care. This collaborative process ensured that the plan remained aligned with the patient’s values and the family’s understanding of his wishes.

Broader adoption of this approach would benefit from institutional and ethical infrastructure, that is, not yet standard. Development of institutional protocols or clinical decision-support tools may help reduce hesitancy regarding the use of sedatives for refractory symptoms during end-of-life care.^15^ Clear institutional protocols addressing the use of anesthetic-depth sedation during palliative extubation including documentation standards, multidisciplinary collaboration, and explicit ethical frameworks distinguishing symptom-directed care from actions intended to hasten death could support consistent practice, and provide legal and ethical clarity for the care teams involved.

The strategy described here may have relevance beyond HE-associated RSE. Any patient with refractory neurological symptoms requiring anesthetic-depth sedation – for example, RSE due to autoimmune encephalitis, hypoxic-ischemic injury, or other metabolic encephalopathies – whose goals of care shift toward comfort-focused care could conceivably benefit from a similar individualized approach. This possibility, however, is speculative and would require further case experience before any general recommendation could be made.

### Limitations

This report is limited by its single-case design, in which direct causality cannot be drawn. The confounding effect of multi-organ failure and complexity of the individual case may also inherently constrain the generalizability of this case. Visible seizure activity, rather than cEEG, was the primary outcome measure during the terminal phase, as continuous monitoring was not maintained through extubation.

## Conclusion

Palliative extubation while continuing propofol and midazolam infusions may represent a viable strategy to maximize comfort in patients with RSE due to HE, though this conclusion is necessarily limited due to the experience of a single case. This case illustrates that standard end-of-life extubation protocols may require deliberate modification in the presence of refractory neurological symptoms. By maintaining therapeutic infusions to prevent agonal seizures, clinicians can meaningfully maximize patient comfort and preserve dignity during the dying process. These observations are exploratory and hypothesis-generating; further case experience and studies are needed before this approach can be more broadly recommended. Multidisciplinary collaboration and transparent family-centered decision-making are essential to ethically navigate these complex situations and deliver individualized, symptom-directed palliative care.

## Supplemental Material

sj-docx-1-sco-10.1177_2050313X261468910 – Supplemental material for Continuation of sedative infusions during palliative extubation for refractory status epilepticus in hepatic encephalopathy: A case report Supplemental material, sj-docx-1-sco-10.1177_2050313X261468910 for Continuation of sedative infusions during palliative extubation for refractory status epilepticus in hepatic encephalopathy: A case report by Liam D. Ferreira and Shailaja Hayden in SAGE Open Medical Case Reports
