# Supplementary material for: Continuation of sedative infusions during palliative extubation for refractory status epilepticus in hepatic encephalopathy: A case report
Source: SAGE Open Med Case Rep. 2026 Jul 18;14:2050313X261468910. doi: 10.1177/2050313X261468910 (PMC13380675; doi:10.1177/2050313X261468910)
Supplement: sj-docx-1-sco-10.1177_2050313X261468910 – Supplemental material for Continuation of sedative infusions during palliative extubation for refractory status epilepticus in hepatic encephalopathy: A case report [file sj-docx-1-sco-10.1177_2050313X261468910.docx]

**CARE Checklist**

Table 2. CARE (CAse REport) Checklist

| **#** | **CARE Item** | **Reported in Manuscript** | **Location** |
| --- | --- | --- | --- |
| 1 | **Title** | Identifies the article as a case report. Title includes 'A Case Report'. ✓ | Title |
| 2 | **Key Words** | Palliative extubation, status epilepticus, hepatic encephalopathy, refractory status epilepticus. ✓ | Manuscript keywords (submission system) |
| 3 | **Abstract** | Unstructured abstract summarizing background, case, and conclusion. ✓ | Abstract |
| 4 | **Introduction** | Background and clinical significance described in Introduction. ✓ | Introduction,  paragraphs 1-2 |
| 5a | **Patient Information** | 53-year-old male. De-identified. Chief complaint: abdominal pain, nausea, vomiting. Relevant history: alcohol-related cirrhosis (Child-Pugh C), metastatic HCC, SBO from diaphragmatic hernia. ✓ | Case Description, paragraph 1 |
| 5b |  | Co-morbidities: end-stage liver disease, HCC, AKI, lactic acidosis. ✓ | Case Description, paragraphs 1-2 |
| 5c |  | Relevant family history: not applicable. ✓ | Case Description |
| 5d |  | Relevant psychosocial history: alcohol use disorder. ✓ | Case Description |
| 6 | **Clinical Findings** | Neurological: completely obtunded; right arm and bilateral myoclonic epileptiform movements. Hemodynamic: BP nadir 40s/20s mmHg; norepinephrine-dependent. Ammonia 601 µmol/L; lactate 10 mmol/L. ✓ | Case Description, paragraphs 2-3 |
| 7 | **Timeline** | Hospital day-by-day timeline table provided in Case Description. ✓ | Table 1 |
| 8a | **Diagnostic Assessment** | Diagnosis: RSE secondary to HE precipitated by SBO from right-sided diaphragmatic hernia in setting of Child-Pugh C cirrhosis and metastatic HCC. ✓ | Case Description, paragraph 1 |
| 8b |  | Diagnostic investigations: cEEG (electroclinical SE, absent reactivity); CT head (stroke/herniation evaluation); metabolic labs. ✓ | Case Description, paragraph 4 |
| 8c |  | Prognostic characteristics discussed in Discussion. ✓ | Discussion |
| 9a | **Therapeutic Interventions** | Antiseizure: levetiracetam 4 g IV load → 750 mg BID; lacosamide 200 mg IV load → 100 mg q12h; midazolam infusion (up to 40 mg/hr); propofol infusion (up to 30 mcg/kg/min); ketamine infusion (20 mcg/kg/min). Hyperammonemia: lactulose enemas, CRRT. Hemodynamic: norepinephrine up to 0.43 mcg/kg/min. ✓ | Case Description paragraph 3 |
| 9b |  | Palliative: vasopressor withdrawal, palliative extubation with continuation of propofol (20 mcg/kg/min) and midazolam (30 mg/hr). ✓ | Case Description paragraph 5 |
| 10a | **Follow-up and Outcomes** | Patient died peacefully without visible seizure activity. ✓ | Case Description paragraph 5 |
| 10b |  | Adverse and unanticipated events: propofol-induced hypotension necessitating vasopressor support; PRIS monitoring discussed. ✓ | Case Description paragraph 3 |
| 11a | **Discussion** | Strengths and limitations of this approach discussed. ✓ | Discussion |
| 11b |  | Relevant medical literature cited and discussed. ✓ | Discussion |
| 11c |  | Rationale for clinical decisions provided (cEEG guidance, pharmacokinetic considerations, multidisciplinary ethics). ✓ | Discussion  paragraph 1-3 and 6 |
| 12 | **Patient Perspective** | Patient was unable to communicate wishes directly due to obtundation. Family (wife) acted as surrogate. Family wishes regarding absence of endotracheal tube at time of death are documented. Written informed consent for publication obtained from patient's wife. ✓ | Case Description paragraph 5,  Disclosures |
| 13 | **Informed Consent** | Written informed consent obtained from patient's wife for publication of this case report. ✓ | Disclosures |
